# Supplementary material for: Bladder Cancer Extracellular Vesicles Elicit a CD8 T Cell-Mediated Antitumor Immunity
Source: Int J Mol Sci. 2022 Mar 8;23(6):2904. doi: 10.3390/ijms23062904 (PMC8949613; doi:10.3390/ijms23062904)
Supplement: Supplementary file 1 [file ijms-23-02904-s001.zip › ijms-1594655-supplementary.pdf]

## Supplementary Figure

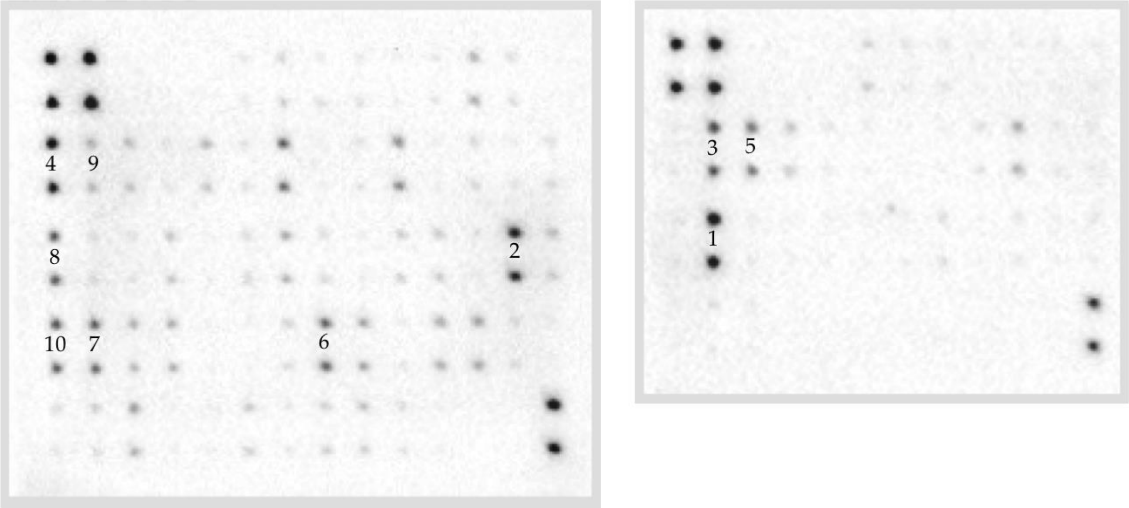


**Supplementary Figure 1. MB49-EVs contain pro-inflammatory cytokines.**
Images of membranes used for the identification of cytokines contained in MB49-EVs as detected via an anti-cytokine antibody array. Indicated numbers correspond to cytokines listed in Figure 5E.

## Supplementary Tables

**Supplementary Table 1. Identified immune system-related proteins in MB49-EVs detected via mass spectrometry.** Total proteins identified in MB49-EVs that participate in the immune system pathways as determined by a PANTHER database analysis are listed. Abundance was calculated by the mass spectrometry analysis as the sum of the peak areas for each peptide that could be integrated for the given protein.

| PANTHER Immune System Pathways | Protein ID | Abundance | Protein ID | Abundance |
|---|---|---|---|---|
| Inflammation Mediated by chemokine and cytokine signaling | Myosin-9 | 179434.70 | Mylk | 1671.50 |
| | Actin-b | 71978.80 | Camk2g | 1608.30 |
| | Integrin beta-1 | 66428.70 | Pak3 | 1600.20 |
| | Collagen 12-a1 | 34064.80 | Itpr1 | 1489.80 |
| | Collagen 6-a1 | 32229.90 | Pak4 | 1371.20 |
| | Gnai-2 | 29601.30 | Rhoc | 1282.40 |
| | Cdc42 | 28249.40 | Gng5 | 1265.50 |
| | Collagen 2-a2 | 25773.10 | Plcd3 | 1203.60 |
| | Rac1 | 21712.70 | Arpc5l | 1189.90 |
| | Rras2 | 18753.60 | Akt2 | 1123.20 |
| | Arpc2 | 15770.00 | Kras | 1093.90 |
| | Arpc4 | 15293.80 | Nfkb2 | 1051.10 |
| | RhoA | 15040.80 | Raf1 | 1037.70 |
| | Camk2d | 13540.40 | Stat6 | 729.40 |
| | Mapk3 | 12887.40 | Rela | 656.40 |
| | Acta2 | 9230.30 | Nfkb1 | 600.60 |

| | | | | |
|---|---|---|---|---|
| | Rhog | 9092.90 | Gnao1 | 568.90 |
| | Rras | 7974.20 | Stat3 | 429.00 |
| | Gnai3 | 7648.40 | Stat1 | 342.30 |
| | Arpc1b | 6673.90 | Ptk2b | 338.10 |
| | Arpc5 | 5411.70 | Akt1 | 327.40 |
| | Nras | 4856.40 | Pla2g4a | 264.60 |
| | Cask | 4472.20 | Vwf | 260.40 |
| | Itga2 | 4113.80 | Grk2 | 214.50 |
| | Grb2 | 3951.90 | Nfkbia | 181.00 |
| | Arpc3 | 3352.40 | Itpr3 | 139.80 |
| | Prkacb | 3017.20 | Itgb7 | 120.70 |
| | Gng12 | 2684.80 | Plcl2 | 102.40 |
| | Ptgs1 | 1870.10 | Ikbkb | 54.80 |
| | Myh10 | 1721.60 | Adcy6 | 45.20 |
| T cell activation | Cdc42 | 28249.40 | Map2k1 | 1168.40 |
| | Rac1 | 21712.70 | Akt2 | 1123.20 |
| | Mapk3 | 12887.40 | Nfkb2 | 1051.10 |
| | B2-m | 8313.40 | Raf1 | 1037.70 |
| | CD80 | 5693.70 | Nfkb1 | 600.60 |
| | Hras | 5658.60 | Akt1 | 327.40 |
| | Nras | 4856.40 | Map2k2 | 311.80 |
| | Grb2 | 3951.90 | Ppp3cb | 226.30 |
| | Ppp3ca | 2530.60 | Nfkbia | 181.00 |
| | Pak3 | 1600.20 | Ptprc | 176.70 |
| | Itpr1 | 1489.80 | Ikbkb | 54.80 |
| | CSK | 1397.90 | | |
| B cell activation | Rac1 | 21712.70 | Raf1 | 1037.70 |
| | Lyn | 17905.60 | Nfkb1 | 600.60 |
| | Mapk3 | 12887.40 | Frk | 450.90 |
| | Ighm | 9455.30 | Map2k2 | 311.80 |
| | Hras | 5658.60 | Map3k2 | 248.90 |
| | NRas | 4856.40 | Ppp3cb | 226.30 |
| | Grb2 | 3951.90 | Mapk12 | 186.40 |
| | Ppp3ca | 2530.60 | Nfkbia | 181.00 |
| | Itpr1 | 1489.80 | Ptprc | 176.70 |
| | Map2k1 | 1168.40 | Itpr3 | 139.80 |
| | Nfkb2 | 1051.10 | Ikbkb | 54.80 |
| Toll-like receptor signaling | Mapk3 | 12887.40 | Rela | 656.40 |
| | Tollip | 3813.10 | Nfkb1 | 600.60 |
| | Ube2v1 | 3668.70 | Map2k4 | 386.30 |
| | Ube2n | 2148.00 | Map2k2 | 311.80 |
| | Map2k1 | 1168.40 | Nfkbia | 181.00 |
| | Map2k3 | 1147.70 | IL-1rak4 | 69.60 |
| | Nfkb2 | 1051.10 | Ikbkb | 54.80 |
| Interleukin signaling | Mapk3 | 12887.40 | Gsk3b | 777.60 |
| | Rps6ka3 | 4884.90 | Stat6 | 729.40 |
| | Nras | 4856.40 | Stat3 | 429.00 |
| | Grb2 | 3951.90 | Stat1 | 342.30 |
| | Stat5a | 1765.40 | Akt1 | 327.40 |
| | Akt2 | 1123.20 | mTOR | 115.90 |
| | Raf1 | 1037.70 | Ikbkb | 54.80 |

| | | | | |
|---|---|---|---|---|
| Interferon-gamma signaling | Mapk3 | 12887.40 | Stat1 | 342.30 |
| | Ptpn11 | 1019.70 | Mapk12 | 186.40 |

**Supplementary Table 2. Cytokines in MB49-EVs Analyzed via Cytokine Antibody Array.** Densitometric analyses was performed using the ImageJ software to measure the intensity of staining for detected cytokines spots. The intensity of detected spots was used to determine their normalized abundance as indicated in the manufacturer's instructions.

| Cytokine | Abundance | Cytokine | Abundance | Cytokine | Abundance |
|---|---|---|---|---|---|
| Osteopontin | 43.69 | G-CSF | 5.57 | VCAM-1 | 2.23 |
| LIX | 31.45 | SCF | 5.48 | NEG | 2.20 |
| ICAM-1 | 27.03 | CTACK | 5.45 | IGF-BP-5 | 2.19 |
| Eotaxin-2 | 24.85 | IL9 | 5.30 | VEGF R1 | 2.17 |
| IGFBP-2 | 17.54 | MMP-2 | 5.25 | IL1-beta | 2.14 |
| MIP-2 | 15.79 | VEGF R2 | 5.13 | I-TAC | 1.92 |
| MCP-1 | 15.75 | IL3 Rb | 4.91 | CXCL16 | 1.65 |
| IL4 | 14.34 | Leptin R | 4.78 | CD30/TNFRSF8 | 1.50 |
| FAS ligand | 13.84 | IL2 | 4.59 | MIP-3-alpha | 1.39 |
| Lymphotactin | 13.71 | GITR | 4.44 | TIMP-1 | 1.35 |
| IGF-BP-3 | 13.47 | MDC | 4.42 | VEGF | 1.32 |
| L-Selectin | 12.67 | Flt-3 Ligand | 4.23 | TECK | 1.20 |
| Fractalkine | 12.08 | TPO | 4.17 | TROY | 1.18 |
| P-Selectin | 11.95 | DPPIV/CD26 | 3.81 | CD30L | 1.13 |
| IL1-alpha | 10.98 | MIP-1-gamma | 3.59 | IL10 | 1.08 |
| Lungkine | 10.17 | TARC | 3.55 | axl | 1.07 |
| MMP-3 | 9.22 | Dtk | 3.53 | sTNF RI | 1.05 |
| IL5 | 8.64 | TNF-alpha | 3.48 | IL17 | 0.96 |
| PF4 | 8.59 | IL3 | 3.44 | CRG-2 | 0.94 |
| RANTES | 8.54 | IL12-p40/p70 | 3.31 | MIP-1-alpha | 0.92 |
| MIP-3-beta | 8.21 | Fcg RIIB | 3.27 | Shh-N | 0.85 |
| MCP-5 | 8.02 | KC | 3.20 | IGF-II | 0.77 |
| TCA-3 | 7.66 | sTNF RII | 3.18 | MIG | 0.69 |
| GM-CSF | 7.09 | Osteoporotegerin | 3.17 | CD40 | 0.61 |
| M-CSF | 6.39 | IGF-BP-6 | 2.87 | TSLP | 0.53 |
| IGF-I | 6.39 | IFN-gamma | 2.75 | Pro-MMP-9 | 0.39 |
| HGF R | 6.33 | E-Selectin | 2.71 | Eotaxin | 0.25 |
| IL6 | 6.10 | SDF-1-alpha | 2.63 | IL-7 | 0.11 |
| bFGF | 6.05 | BLC | 2.53 | IL-15 | 0.07 |
| Leptin | 6.03 | TIMP-2 | 2.39 | IL-17B R | 0 |
| IL12-p70 | 5.70 | IL13 | 2.38 | Resistin | 0 |
| VEGF R3 | 5.60 | Thymus CK-1 | 2.24 | TRANCE | 0 |
